# A Scalable, Web-Based Platform for Proteomics Data Processing, Result Storage and Analysis

**DOI:** 10.1021/acs.jproteome.4c00871

**Published:** 2025-02-21

**Authors:** Markus Schneider, Daniel P. Zolg, Patroklos Samaras, Samia Ben Fredj, Dulguun Bold, Agnes Guevende, Alexander Hogrebe, Michelle T. Berger, Michael Graber, Vishal Sukumar, Lizi Mamisashvili, Igor Bronsthein, Layla Eljagh, Siegfried Gessulat, Florian Seefried, Tobias Schmidt, Martin Frejno

**Affiliations:** 1 MSAID GmbH, Garching b. München 85748, Germany; 2 MSAID GmbH, Berlin 13347, Germany

**Keywords:** proteomics, platform, pipeline, CHIMERYS, compute infrastructure, data processing, cloud, AWS, scalable, SaaS

## Abstract

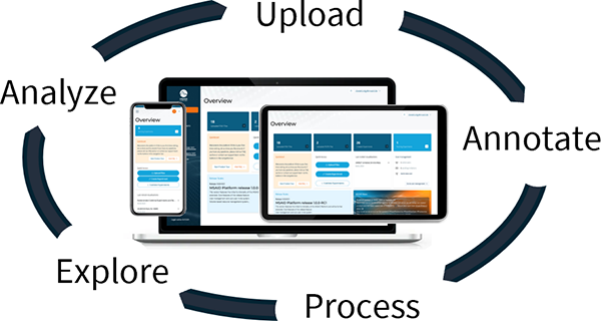

The exponential increase in proteomics data presents critical challenges for conventional processing workflows. These pipelines often consist of fragmented software packages, glued together using complex in-house scripts or error-prone manual workflows running on local hardware, which are costly to maintain and scale. The MSAID Platform offers a fully automated, managed proteomics data pipeline, consolidating formerly disjointed functions into unified, API-driven services that cover the entire process from raw data to biological insights. Backed by the cloud-native search algorithm CHIMERYS, as well as scalable cloud compute instances and data lakes, the platform facilitates efficient processing of large data sets, automation of processing via the command line, systematic result storage, analysis, and visualization. The data lake supports elastically growing storage and unified query capabilities, facilitating large-scale analyses and efficient reuse of previously processed data, such as aggregating longitudinally acquired studies. Users interact with the platform via a web interface, CLI client, or API, providing flexible, automated access. Readily available tools for accessing result data include browser-based interrogation and one-click visualizations for statistical analysis. The platform streamlines research processes, making advanced and automated proteomic workflows accessible to a broader range of scientists. The MSAID Platform is globally available via https://platform.msaid.io.

## Introduction

Proteomics is an indispensable technology for the comprehensive identification and quantification of proteins, which are pivotal for understanding cellular functions and disease mechanisms. Over the past decade, there have been substantial advancements in the key components of proteomic workflows, including sample preparation techniques, liquid chromatography (LC), and mass spectrometry (MS) instrumentation.^1,2^ These improvements, particularly the advent of fast-scanning mass spectrometers, have significantly enhanced the sensitivity, comprehensiveness, and throughput of proteomic analyses.^3^ Consequently, researchers can now conduct large-scale proteomic studies that generate an unprecedented volume of raw data. However, this surge in data production presents substantial challenges for data processing pipelines generating protein identifications and associated quantitative information. The growing size of raw and result data and sheer number of mass spectrometry measurements that can be performed in a short period of time regularly exceed the capabilities of conventional on-premises compute infrastructure, particularly with respect to the demanded processing power and storage space.^4,5^

In parallel, recent years have seen a fast-paced development and improvement of software for proteomics data processing, fueled by the introduction of deep learning-based prediction of peptide properties.^6−9^ Academic software, such as MSFragger^10^ and rescoring concepts like Prosit,^6^ MSBooster,^11^ MS^2^Rescore,^12^ EncyclopeDIA,^13^ DeepDIA,^14^ AlphaDIA,^15^ DIA-NN,^16^ and commercial products like INFERYS,^17^ Spectronaut^18^ and CHIMERYS,^19^ have pushed the boundaries of data extraction, enabling deeper insights into complex proteomics data sets by leveraging fragment ion intensities.

Together, the combination of instrumentation and more sensitive algorithms allows researchers to generate protein profiles to an unprecedented depth and throughput. However, in contrast to today’s streamlined sample workflows in the wet lab leading up to the mass spectrometer, the data flow from the acquired raw data to the extraction of biological insights remains fragmented and often inefficient. Laboratories are confronted with a range of computational challenges as they attempt to process and analyze their data: frequently encountered manual workflows are not only time-consuming but also prone to errors and inconsistencies, particularly when repetitive tasks are involved. A data pipeline might involve the manual transfer of raw files from the acquisition computer to a storage medium, which may be a local personal computer, a laptop, or, in some cases, a network-attached storage (NAS) system, rarely a cloud-based service. Subsequent processing of the data with a proteomic search engine involves the use of local consumer hardware such as personal computers or, in some cases, high-performance servers. This reliance on local hardware inherently limits the scalability of proteomic studies, as the computational demands of large-scale analyses often exceed the capabilities of on-premises infrastructure or the scalability of the software package itself. Once processed, the results are usually manually moved to user- or project-specific directories and shuffled between different storages to avoid disk exhaustion, causing confusion and parallel systems of data organization, limiting accessibility to other researchers or data mining. The level of subsequent data interrogation and interpretation varies drastically depending on the researcher’s skill set, with approaches ranging from basic spreadsheet analyses to more sophisticated bioinformatics tools (e.g., Perseus^20^) or scripting languages. Dedicated statistics and visualization suites like MSstats^21^ and Mass Dynamics^22^ can aid non-bioinformaticians in drilling down on their biological question, but also present standalone solutions, adding to a fragmented tooling landscape. The described patchwork of disconnected local infrastructures and applications create highly redundant work streams, render it difficult to automate processes, risk loss of data integrity, and hinder the generation of reproducible results. While custom scripts and pipelines might attenuate the manual labor in the process, these are often hastily developed, lack robustness and are costly to maintain long-term. Bespoke in-house solutions frequently do not scale well with the size of projects, growing infrastructure, or team size, due to the effort of coordinating limited resources in an exponentially growing data environment.

To streamline the proteomic data workflow, we introduce the MSAID Platform—a comprehensive, managed, and cloud-based one-stop-shop for proteomics. It facilitates data handling, storage and analysis, allowing researchers to focus on scientific questions of interest. By leveraging the scalability and flexibility of cloud computing, this platform eliminates the limitations of local hardware, enabling researchers to run experiments at any time, without having to worry about resource limitations and processing vast data sets automatically, efficiently, and reproducibly. Through its application programming interface (API)-based design, advanced users retain the ability to tailor workflows to their needs, facilitating seamless integration with existing or new tools, providing a “best of both worlds” approach if desired.

## Materials and Methods

**Figure 1:**
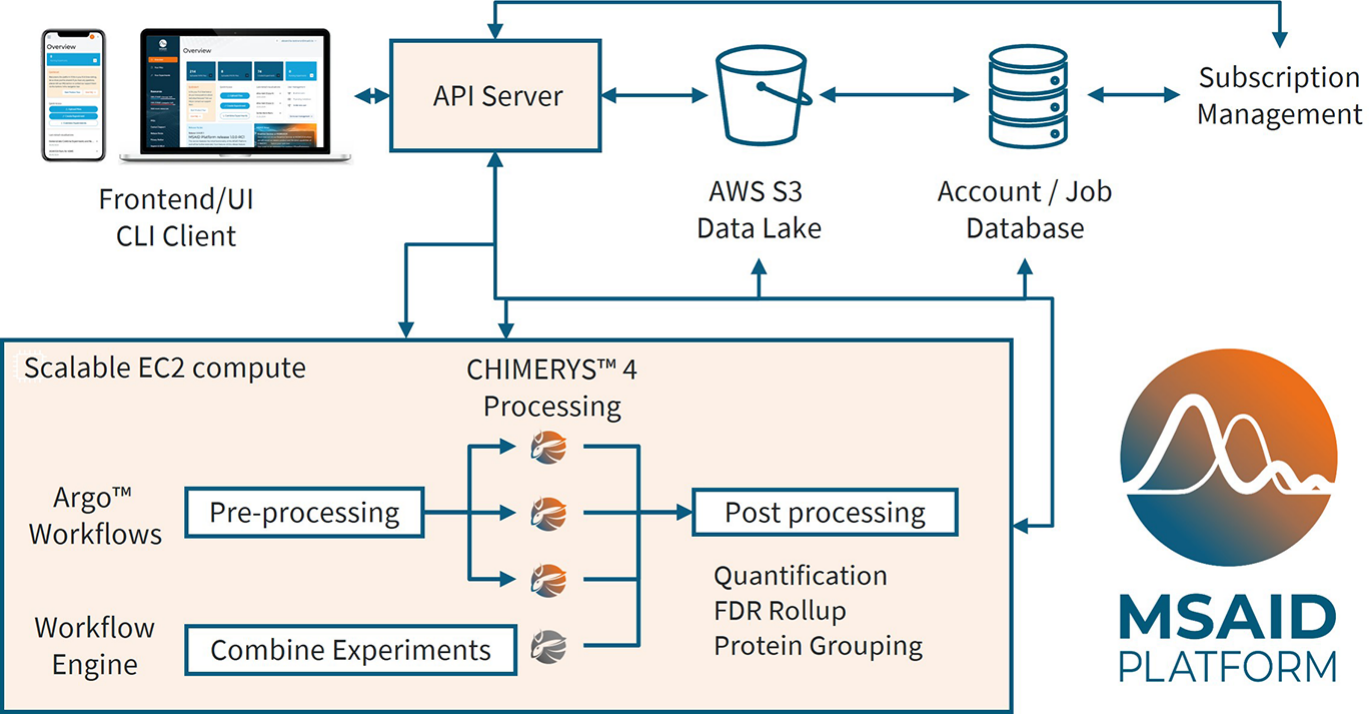
The MSAID Platform for proteomics comprises a cloud-native, microservices-based architecture, orchestrated by Kubernetes. It is hosted on Amazon Web Services (AWS), utilizing Elastic Kubernetes Service (EKS). The platform supports multiple interfaces, including a web interface, command-line interface (CLI), and an application programming interface (API) access for seamless user interaction. Uploaded raw and fasta files are stored as on AWS Simple Storage Service (S3). A relational database (RDS) manages the data lake and meta-attributes for files and processing jobs. Scalable CHIMERYS workflows for data-dependent acquisition (DDA), data-independent acquisition (DIA) or parallel reaction monitoring (PRM) data processing can be executed on AWS Elastic Compute Cloud (EC2) instances. The platform offers the option to continuously acquire and search raw files, while raw file overarching postprocessing like protein grouping are performed later without researching the data. Result data is systematically stored as parquet files and can be interactively explored and visualized in the browser or downloaded via browser, CLI, or API for further exploration.

The MSAID Platform is designed as a cloud-native solution, employing a microservices architecture orchestrated by Kubernetes to ensure both scalability and flexibility across various computational tasks (Figure 1). In its current inception, it is hosted on Amazon Web Services (AWS) but is compartmentalized for future deployment into other cloud service providers or a local server solution. Platform services and compute resources are deployed using an AWS Elastic Kubernetes Service (EKS) cluster, with automated infrastructure management facilitated by Terraform and Helm. User management, including authentication and authorization, is handled through AWS Cognito, incorporating multifactor authentication to ensure secure access and compliance with data protection protocols. Centralized control of the platform’s operations is achieved through an API server, which governs all aspects of data handling, processing, and user interactions (Supporting Figure S1). The platform supports multiple interfaces for user interaction: an interactive web interface built with Vue.js provides an accessible and intuitive interface and offers a responsive design for use with personal computers, tablets, and even smartphones; a command-line interface (CLI) caters to users who require automation or scripting capabilities; and direct API access allows for seamless integration with other tools and systems. Data storage within the platform is managed as a data lake on AWS Simple Storage Service (S3), with parquet files as the backing file format. Trino and DuckDB are used to provide a systematic and uniform query layer across experiment results, making use of distributed computation to enable powerful online analyses without downloading large result files. The primary user of an account may invite other users to share a single workspace, enabling collaboration. Two-factor authentication is available to protect user accounts. Data locality is fixed to European data centers for European users, with the architecture designed to accommodate future expansion to additional regions. AWS Relational Database Service (RDS) is utilized to store data attributes, metadata, and details of processing jobs, though the processed results themselves are stored separately. Data processing workflows are orchestrated by Argo Workflows, which manages the distribution and execution of tasks on Elastic Compute Cloud (EC2) instances. The platform allows users to search data with the CHIMERYS search algorithm, which is described in detail in Frejno et al., 2024.^19^ The platform offers multiple avenues for data interaction, including file download via the browser, a simple web-based data browser, as well as statistical testing and interactive visualization capabilities facilitated by the Vega library (https://vega.github.io/vega/).

## Results

**Figure 2:**
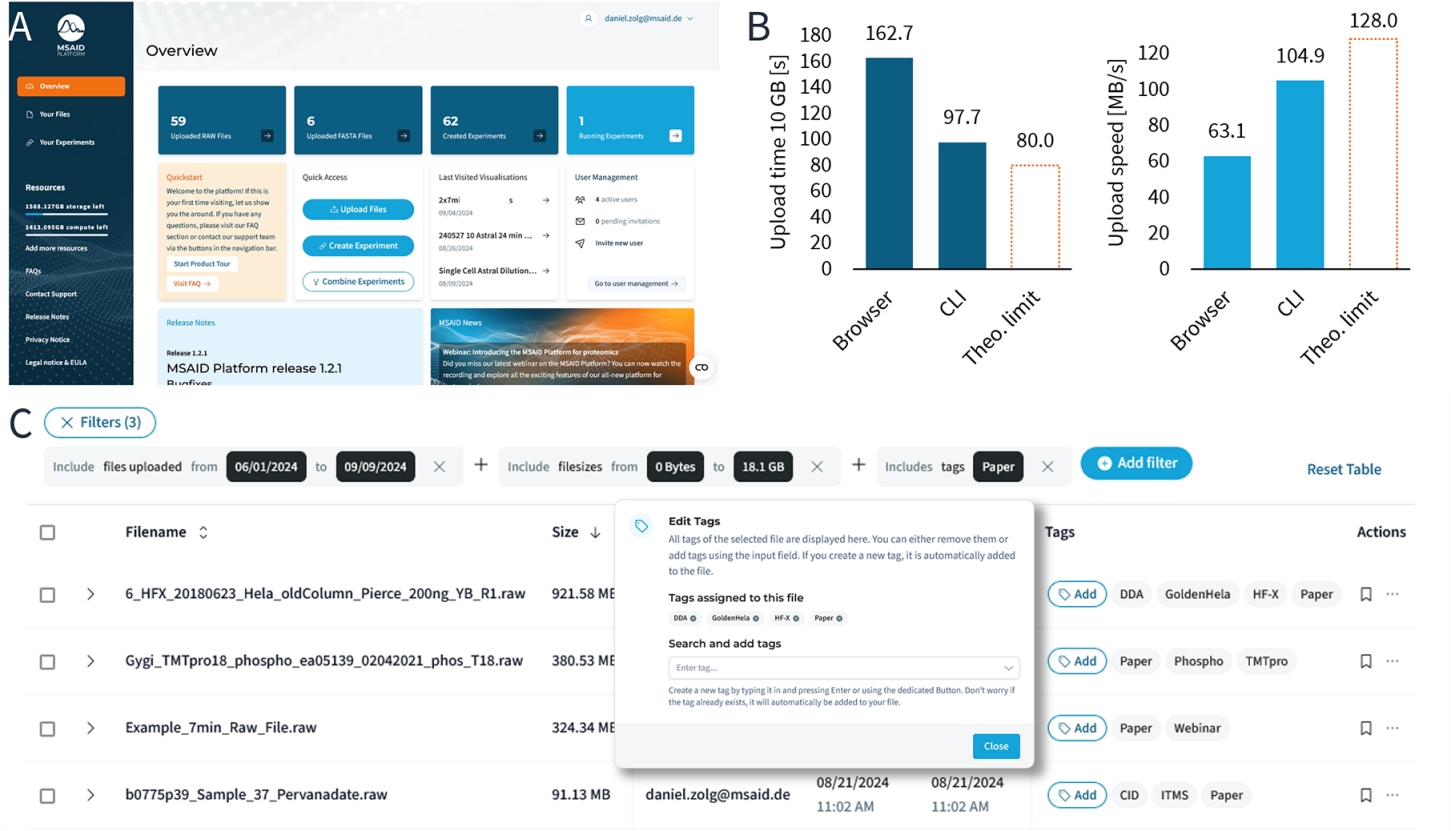
(A) The welcome screen of the platform presents key statistics of the user account, including the number of running searches, quick links to the latest triggered experiments and the available processing quota. (B) Speed comparison of the browser-based (Firefox v130.0) and CLI-based 10 GB raw data upload into the AWS S3 data lake using a Windows Server 2022 server connected with a 1 Gbit/s uplink. Theoretical limit is determined as the maximum achievable throughput of a 1 Gbit/s uplink. (C) Data management and organization are facilitated by adding free text tags. Both tags and auto-generated metadata can be used in no-code queries for data retrieval. Images reproduced with permission from MSAID.

One of the major goals of the platform is to further democratize and streamline access to proteomic data processing, to enable wet-lab scientists and non-bioinformatic experts to work with large-scale data and obtain results efficiently. The landing page of the platform aids the user experience by providing an overview of the most important metrics, like file statistics, last visited experiments, available processing quotas and user management (Figure 2A). The first step to a proteomic processing job (here called Experiments), is to upload data for processing.

Proteomic data size has grown drastically over time, with several petabytes of data being deposited into repositories of the ProteomeXchange consortium^23^ every year. Today, individual studies may encompass several thousand raw files and terabytes of raw data. To accommodate the efficient and secure upload of such large datasets, the platform offers two distinct upload methods: the web interface offers simple interaction with the S3 data lake. The upload process is optimized for reliability and speed, with functionalities such as data chunking, integrity checks, and the ability to automatically retry and resume partial uploads in case of interruptions. Uploads reach speeds of >60 MB/s via common web browsers without the need for plugins or dedicated software (Figure 2B). The robustness of this method has been validated through extensive testing and handles single files >50 GB without inducing noticeable load on the browser.

For scientists requiring more flexibility or those managing large volumes of data regularly, a CLI client is available for Windows, Linux, and macOS. The CLI client offers similarly secure, error-resilient uploading but with enhanced functionality and speed. Notably, the CLI client includes a ‘watch’ command that allows continuous monitoring of a specified folder using freely configurable regular expressions to include and exclude expressions, such as “HeLa” or “QC”. Upon completion of raw data acquisition, files matching these expressions are automatically uploaded. This feature is particularly advantageous for longitudinal studies or quality control applications, where data files are generated repeatedly or over a longer period. The CLI client has been optimized to achieve upload speeds of >100 MB/s on a 1 Gbit/s uplink, rendering the upload of even large studies feasible in just a few hours (Figure 2B).

Data security and compliance are central to the platform’s design; all hosting is performed on AWS, an ISO norm (by the International Organization for Standardization) and CSA STAR (Security, Trust, Assurance, and Risk) program by the CSA Group certified provider. All data are encrypted in transit and at rest and securely stored on S3, benefiting from its inherent redundancy and recovery features. Stringent access control via Access Control Lists (ACLs) ensures that each user’s data is isolated from others, aligning with state-of-the-art security practices and compliance requirements, including the EU General Data Protection Regulation (GDPR).

During and after upload, the platform provides data tagging and metadata management capabilities. Users can tag data with free-text labels, facilitating fully customizable organization and retrieval. This tagging system integrates seamlessly with the platform’s table-based data management feature, allowing users to organize their raw or fasta files and construct powerful no-code filtering queries based on various data attributes within the browser (Figure 2C). Uploaded fasta protein databases can be associated with parse rules to ensure proper extraction of protein names, gene names, and organisms to cater for the various sources of fasta files.

**Figure 3:**
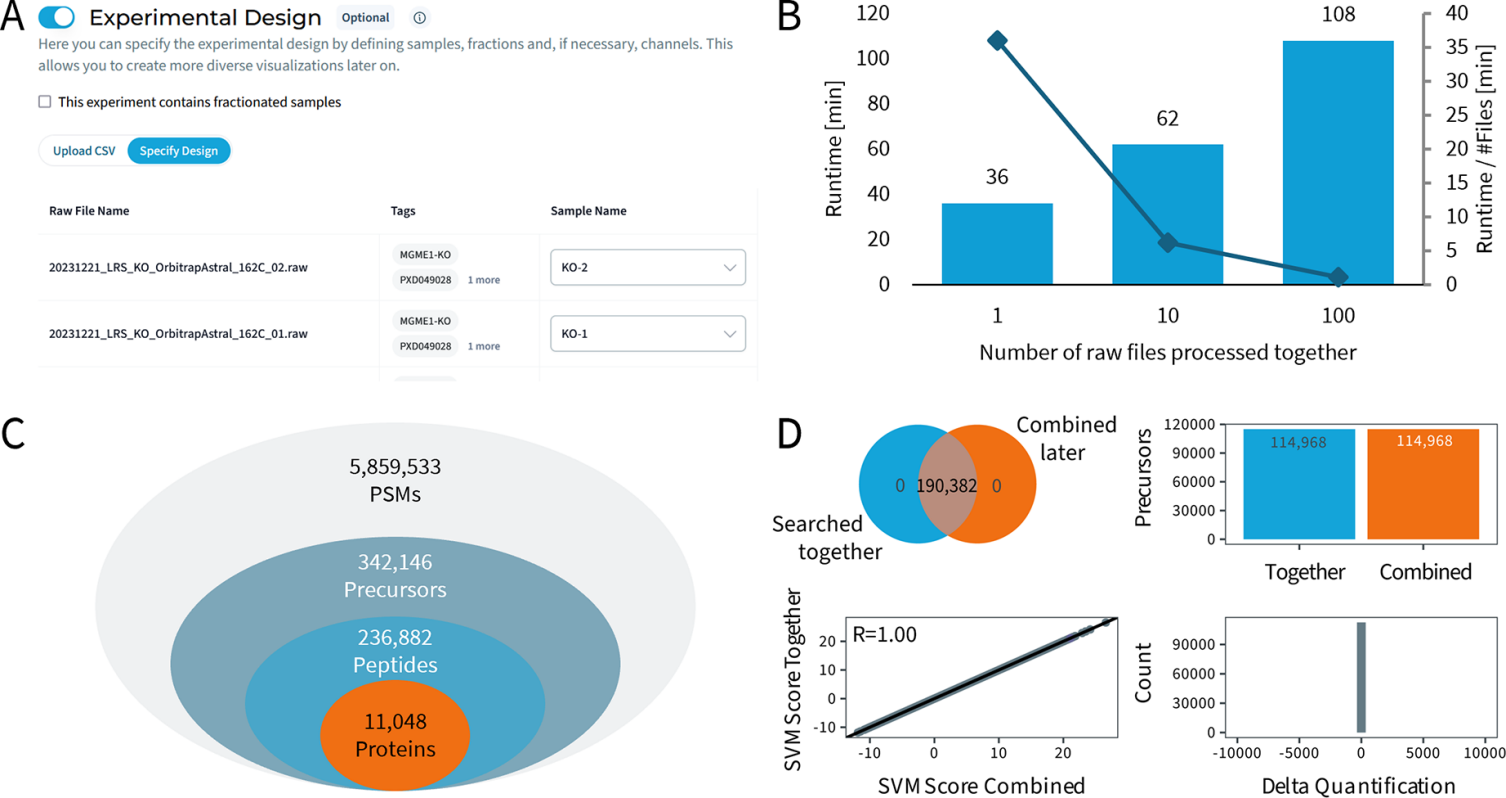
(A) The experimental design acts as an additional layer of meta data annotation. Raw files can be annotated as samples for later visualization. In the case of tandem mass tag (TMT)-labeled samples, the individual channels can be annotated. (B) Runtime comparison of a 1h Q Exactive HF-X HeLa Files run individually and as copies of the same file in parallel. Identification, error control, and quantification were performed across all files. (C) Identification numbers for an offline-fractionated DIA data set acquired with an Orbitrap Astral. Peptide-spectrum matches (PSMs) are represented at 1% file-local false discovery rate (FDR), all other levels at 1% data set-global FDR. Raw data reprocessed from Serrano et al.^24^ (PRIDE data set identifier PXD049028). (D) Comparison of 2 HeLa samples searched together or combined later in postprocessing demonstrates the result identity of longitudinally processed and later aggregated data. Displayed are PSMs at 1% PSM FDR (Venn diagram), unique precursors at 1% precursor FDR (bar chart), a scatterplot of mokapot SVM score of all precursors irrespective of FDR with a Pearson correlation of 1.00 and the delta in precursor quantitation at 1% precursor FDR, all indicating result identity.

Processing of proteomic data is facilitated through an intuitive, multistep wizard that guides users in setting up experiments. This wizard assists browsing, filtering, and selecting input files, making it straightforward for users to initiate their analyses. It also allows recording the experimental design of a study for record keeping and to facilitate later statistical testing and visualization (Figure 3A).

The platform’s design is search engine-agnostic, enabling integration with any search engine that can operate within a Docker container. Currently, the platform is powered by CHIMERYS 4,^19^ with plans to incorporate additional search engines in the future. CHIMERYS is capable of handling Data-Dependent Acquisition (DDA), Data-Independent Acquisition (DIA), and Parallel Reaction Monitoring (PRM) experiments. It operates in a fully spectrum-centric manner, features the deconvolution of chimeric spectra, and incorporates the INFERYS 4 deep-learning model, which provides retention time and fragment ion intensity predictions for the most common post-translational modifications (PTMs) like phosphorylation, acetylation, ubiquitination, cysteine modifications, oxidation, tandem mass tags (TMT), and isotopically labeled amino acids. An in-depth characterization of the CHIMERYS algorithm is available in a separate manuscript.^19^ The processing pipeline also includes comprehensive postprocessing features, such as MS1- and MS2-based quantification via deconvolution and TMT reporter ion-based quantification. During postprocessing, Mokapot^26^ and Picked Protein Group FDR^25^ and are employed for rigorous error control. Processing templates for experiments can be saved by the user to facilitate setting up standardized experiments. The settings can also be exported to directly submit jobs using the CLI client instead of interacting with the graphical user interface (GUI). At the time of writing, CHIMERYS and hence the platform is compatible with all Thermo Scientific mass spectrometers. Compatibility with other vendors and open formats like mzML is expected within the year 2025.

The cloud-native setup allows for the deployment of several hundred compute pods, backed by hundreds of central processing unit (CPU) cores and graphics processing unit (GPU) instances, ensuring that processing remains efficient and fast regardless of the data volume or parallel usage of the platform. Raw files are processed in parallel, with subsequent combination of results of all searches during postprocessing to optimize the overall analysis runtime. Elastic scaling of the platform is achieved using an autoscaler, which analyzes submitted workloads and dynamically acquires or releases computation resources. This strategy keeps the cluster size appropriate to the scheduled compute tasks and ensures efficient processing from low activity times to load spikes. Currently, the cluster can simultaneously spawn >1,000 compute instances, and we are working to expand this capacity by an additional order of magnitude and expand to more than a single datacenter/availability zone to service users across the globe. Performance benchmarks demonstrate the scalability of the platform. Processing a single 1 GB HeLa file including MS1 quantification takes 36 min, while processing 100 files concurrently extends the total time to 108 min (Figure 3B), resulting in 56x faster processing than acquisition time. A published, fractionated Orbitrap Astral DIA data set^24^ comprising 103 GB in size was processed 2.7x faster than acquisition time (198 min processing, 552 min acquisition time), further highlighting the platform’s capability to handle large data sets, even if they are not automatically uploaded and streamed (data not shown). The analysis resulted in 5,859,533 peptide-spectrum matches (PSMs) at 1% file-local FDR, 342,146 precursors, 236,882 peptides and 11,048 protein groups (at 1% dataset-global FDR), underlining the exceptional depth of proteomic profiling that can be achieved nowadays from a single biological sample (Figure 3C).

The platform also supports the efficient reuse of existing data, via combination of previously generated experiments, benefiting quality control (QC) applications and longitudinal data collection and analysis. Users can process each raw file as it becomes available, also through a fully automated workflow via the CLI client. Once a study is completed, experiments processed with compatible settings can be easily combined through a simple wizard in the browser or the CLI. This combination triggers a rerun of the computationally inexpensive postprocessing steps only, including quantification, FDR roll-up, and picked-protein grouping, allowing users to benefit from the thorough analysis of individual files while also obtaining comprehensive results from the entire study without the need for a full search engine run. Due to the deterministic and reproducible results of the processing step, no difference in results (Pearson correlation of R = 1.00) is observed whether data is processed together or combined later (Figure 3D). During the execution of experiments, users can monitor their progress in real-time via the browser. Once an experiment concludes, the platform provides an overview of identified PSMs, peptides, and protein groups, offering immediate insight into the results.

**Figure 4:**
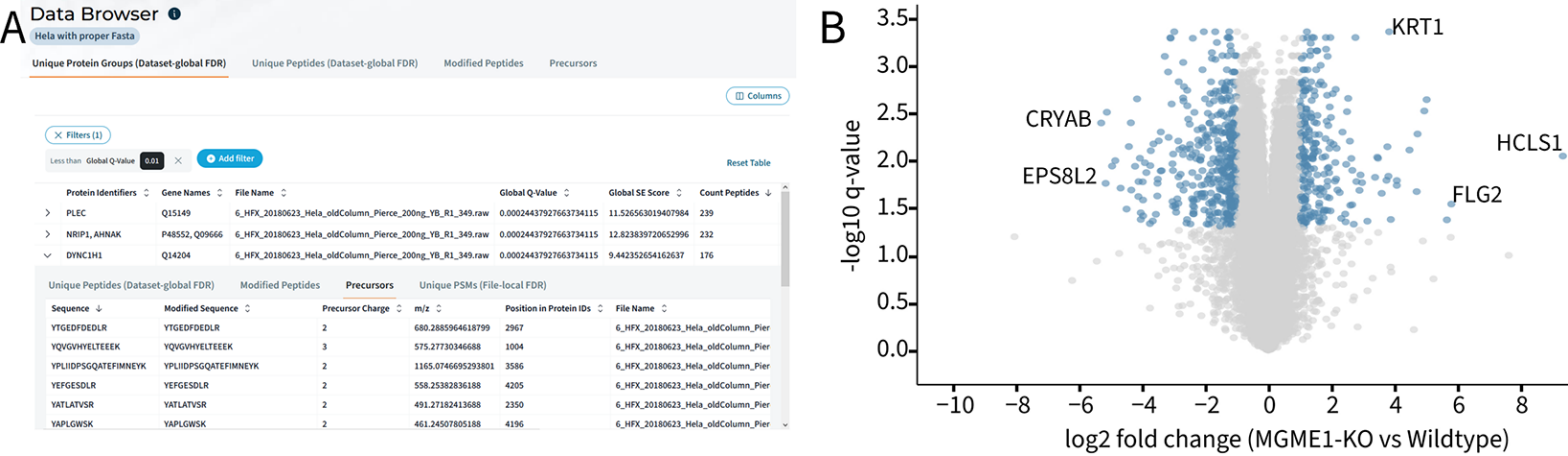
(A) Exploration of results directly in the browser, including nested associations of all contributing data levels (PSMs, precursors, modified peptides, protein groups). (B) Volcano plot on protein group level created within the platform contrasting a CRISPR-Cas9 mitochondrial MGME1 gene Knockout (KO) in human HAP1 cells with wildtype (WT) HAP1 cells. Raw data reprocessed from Serrano et al.^24^ (PRIDE data set identifier PXD049028). A two-sided *t* test was performed for all proteins with complete observation on *n* = 3 replicate single shots for WT and KO. Benjamini–Hochberg was used to calculate false discovery rate (FDR). CHIMERYS processing yields 639 significantly (*q*-value ≤ 0.05) regulated proteins with an absolute fold-change of ≥2.

To allow the user to engage with their results, the platform provides a range of interactive tools. The processed data is systematically stored in a data lake, enabling complex queries across potentially thousands of files. A Trino/DuckDB data lake query layer allows users to retrieve or analyze data directly in the browser (Figure 4A). Tab-Separated Values (TSV) files can be exported and downloaded, providing users complete control over their results for offline storage and processing if desired. File downloads can be fine-tuned with options to apply FDR filtering, formatting and level selection (PSMs, precursors, modified peptides, peptides and protein groups). The platform output is evolving to conform to existing standards (e.g., SDRF^23^) and will soon offer integration with frequently used tools such as Skyline. Additionally, the CLI allows to download the results of submitted jobs directly, enabling users to upload, process, and download results within their pipelines without requiring any interaction with the GUI.

As an alternative to downloading data, users can explore their results online through a data browser that provides an intuitive tabular overview of the full result set set, including advanced filtering and search functionalities backed by Trino’s distributed query engine. This data browser allows users to gain valuable insights into their data before committing to a large download, for example, to quickly determine if proteins of interest have been detected. Nested tables link all evidence levels, facilitating detailed examination of data, such as the quality of all detected PSMs associated with a specific protein.

In addition to allowing access to fully searchable online results, the data lake structure enables scientists to perform statistical testing and visualization directly in the browser. Each experiment includes an interactive, modifiable, and restorable visualization dashboard (Figure 4B). This dashboard offers simple, one-click creation of a variety of customizable plots, such as bar plots for identification numbers and scatter plots visualizing the correlation between files, as well as common tools like UpSet plots, Principal Component Analysis (PCA) and differential expression analysis with Volcano plot visualizations. Both the data points underlying the plot (TSV files) and the plots themselves (vector graphics or Portable Network Graphics [PNGs]) can be downloaded. The plotting capabilities of the platform will expand continuously, aiming to eliminate the need for external analysis tools like R or Python for straightforward data exploration by integrating more functionalities over time.

Overall, we have introduced the first publicly accessible all-in-one Software as a Service (SaaS) platform for proteomics. Our goal was to create an easy-to-use solution for managing and processing proteomic data that can handle swiftly growing volumes of data, while relieving users from the need to buy and manage large compute and storage systems to keep up with the speed of data acquisition. The cloud-native design ensures scalable data upload, management, processing, and result deposition, with features for systematic result exploration and advanced online data interaction directly in the browser. We believe this platform provides a strong foundation, marking the beginning of moving proteomic data processing to the cloud. It empowers researchers by decoupling scientific tasks from the underlying compute and to focus on solving problems instead of spending time managing infrastructure.

## Discussion

The MSAID Platform represents a pioneering effort in the field of proteomics, offering a managed proteomic pipeline and storage solution with an intuitive browser-based interface that eliminates the need for individual laboratories to manage their own infrastructure. This approach significantly lowers entry barriers, particularly for smaller laboratories that may lack the resources to establish and maintain complex data processing pipelines. This contrasts our solution to pipelines like quantms,^4^ which require a self-managed compute environment and only provide a command line interface. By automating the data pipelines from raw data to conclusions, the platform streamlines research processes, making advanced proteomic workflows accessible to a broader range of scientists.

Implementing a cloud-native proteomic workflow addresses a critical need for scalable analyses that keep pace with the rapid growth of data volume fueled by recent developments of faster and more sensitive instruments. A single mass spectrometer running at full efficiency can generate more than a terabyte of raw data within a week, presenting substantial storage and resource challenges that quickly exceed the capacity for local compute clusters, which are not easily scalable. Even batch-processing cloud models struggle with scalability, prolonged transfer times, and lack of integrated storage.

In contrast, the platform offers a private proteomic data lake, enabling users to store and analyze large data sets without hardware constraints. Integrated online workflows eliminate the need for repeated data uploads and downloads, allowing efficient data reuse. To further streamline the workflow, the platform includes tools for automated raw data uploads and result downloads, simplifying the analysis process for researchers. Future developments will leverage the data lake to provide advanced features, such as generating insights from previous experiments, creating downstream analyses, and producing aggregated data views and additional visualizations. Programming libraries for R and Python will offer direct interaction with the results, enabling custom analysis. Additionally, the API will facilitate programmatic access to both experiment-specific and cross-experimental data, ensuring flexibility and integration into diverse research workflows.

The cloud-based nature of the platform may raise concerns regarding security and associated costs. To address these concerns, the platform follows state-of-the-art data handling including encryption and strict ACLs. Further reinforcing the commitment to security, we pursue an ISO27001 certification, which will make it easier to adopt the platform for companies and researchers operating in regulated environments. To provide scientists with the opportunity of exploring the platform, a generous free processing package is available.

Currently, the SaaS solution is fully managed by us, but we are aware of the demand for additional compliance and access management through alternative deployment options. In response, we plan to offer Virtual Private Cloud (VPC) deployments into user-owned cloud accounts, in turn providing enhanced compliance, access control, and data sovereignty. Initially, this will be available for AWS, with future expansion to other cloud providers. While the platform currently relies on AWS services, the core components of the platform are cloud-native technologies not specific to AWS, enabling adaptation to other Kubernetes environments in the future. For example, the S3 data storage can be replaced with any S3 compatible object storage solution like Google Cloud Storage, Azure Blob Storage, or MinIO with reasonable effort. For organizations with existing high-performance computing (HPC) infrastructure or those preferring on-premises solutions, we are also developing a local server deployment option. This approach offers key advantages, including complete data control, offline access, and tailored cost management. It provides a highly viable solution for laboratories operating in sensitive environments. In addition, public funding opportunities often favor one-time hardware and software purchases and have yet to fully adapt to supporting recurring compute costs, even though modern software packages, including essential tools like office suites, are transitioning to SaaS.

SaaS allows for continuous feature delivery and improvement and to quickly patch critical software exploits. To ensure reproducibility, a two-tiered deprecation strategy is followed: Updates to CHIMERYS that change results are released as new minor versions (e.g., 4.1 → 4.2) and remain available for at least one year. Critical security patches may replace earlier versions within the same minor release without affecting results (e.g., 4.1.0 → 4.1.1). This approach balances software security with reproducibility of prior data. While the platform is not intended as a permanent data storage solution at this stage, we plan to introduce data archiving options at a fraction of the cost of S3 storage, reducing the need for local backups. We will also focus on simplifying data integration, including importing data from public proteomic repositories to facilitate a neglected data workflow in the proteomic community: the reuse and reanalysis of the wealth of publicly available and previously analyzed data. In addition, we plan to streamline publishing results obtained on the platform, by e.g. directly uploading data, metadata and results to repositories like PRIDE or allowing a public “view-only” option for obtained results.

Looking ahead, we aim to enhance the platform’s visualization capabilities, driven by user feedback. This includes developing intuitive QC reports and plots and offering diverse views into the underlying MS data (e.g., visualization of quantification traces or a potential Skyline integration), emphasizing our viewpoint that visual inspection of raw data remains crucial and should be taught. While the platform does not yet fully replace offline data analysis, ongoing development aims to close this gap. Our vision is for biologists to focus on interpreting results, such as understanding the Volcano plot on a molecular level, rather than worrying about how to juggle terabytes of experiment data in the process of generating it.

Looking further ahead, we consider the platform as the foundation for large-scale proteomic result generation and exploitation. After addressing the data processing challenges, our focus will shift to advanced data interrogation. This will involve linking with external resources (like PhopshoSitePlus,^27^ UniProt^28,29^ and ProteomicsDB^30,31^), better utilizing insights already available from other tools, and culminates in integrating large language models (LLMs) for conversational data analysis.^32^ Ultimately, we aim to help researchers generate hypotheses to follow up on the ever-growing volume of data, made possible by an integrated workflow from raw files to results and the systematic storage provided by the platform.

In summary, we are advancing into the cloud age of proteomics and project that the MSAID Platform will become an essential tool with a low entry barrier for researchers. Our long-term vision is making proteomic research more accessible and efficient for the expert and non-expert proteomic community.

## Data Availability

The platform can be tested free of charge after registering at https://platform.msaid.io

## Supporting Information Available

(Figure S1) Structure of the MSAID Platform API (PDF)

## Abbreviations

API: application programming interface
AWS: Amazon Web Services
ACL: access control list
CLI: command-line interface
CPU: central processing unit
CSA STAR: CSA Group created Security, Trust, Assurance, and Risk (STAR) Program
EC2: AWS elastic compute cloud
EKS: AWS elastic kubernetes service
FDR: false discovery rate
GDPR: EU General Data Protection Regulation
GUI: graphical user interface
GPU: graphics processing unit
HPC: high-performance computing
ISO: International Organization for Standardization
LC: liquid chromatography
LLM: large language model
MS: mass spectrometry
NAS: network-attached storage
PCA: principal component analysis
PNG: portable network graphic, file format
PSM: peptide-spectrum match
QC: quality control
R: R statistical computing language
RAW: raw data, file format
RDS: AWS relational database service
S3: AWS simple storage service
SaaS: software as a service
SDK: software development kit
TMT: tandem mass tag
TSV: tab-separated values, file format
VPC: virtual private cloud
