# Supplementary material for: A Scalable, Web-Based Platform for Proteomics Data Processing, Result Storage and Analysis
Source: J Proteome Res. 2025 Feb 21;24(3):1241–9. doi: 10.1021/acs.jproteome.4c00871 (PMC11894649; doi:10.1021/acs.jproteome.4c00871)
Supplement: Supplementary file 1 — pr4c00871_si_001.pdf [file pr4c00871_si_001.pdf]

Supporting Information

A scalable, web-based platform for proteomics data processing, result storage and analysis

Authors:

Markus Schneider[1$], Daniel P. Zolg[1$], Patroklos Samaras[1], Samia Ben Fredj[1], Dulguun Bold[1], Agnes Guevende[1], Alexander Hogrebe[2], Michelle T. Berger[1], Michael Graber[1], Vishal Sukumar[1], Lizi Mamisashvili[1], Igor Bronsthein[2], Layla Eljagh[1], Siegfried Gessulat[2], Florian Seefried[1], Tobias Schmidt[1] and Martin Frejno[1*]

[$] Contributed equally

Affiliations:

[1] MSAID GmbH, 85748 Garching b.München, Germany

[2] MSAID GmbH, 13347 Berlin, Germany

Table of contents:

Supporting Figure S1: Structure of the MSAID Platform API

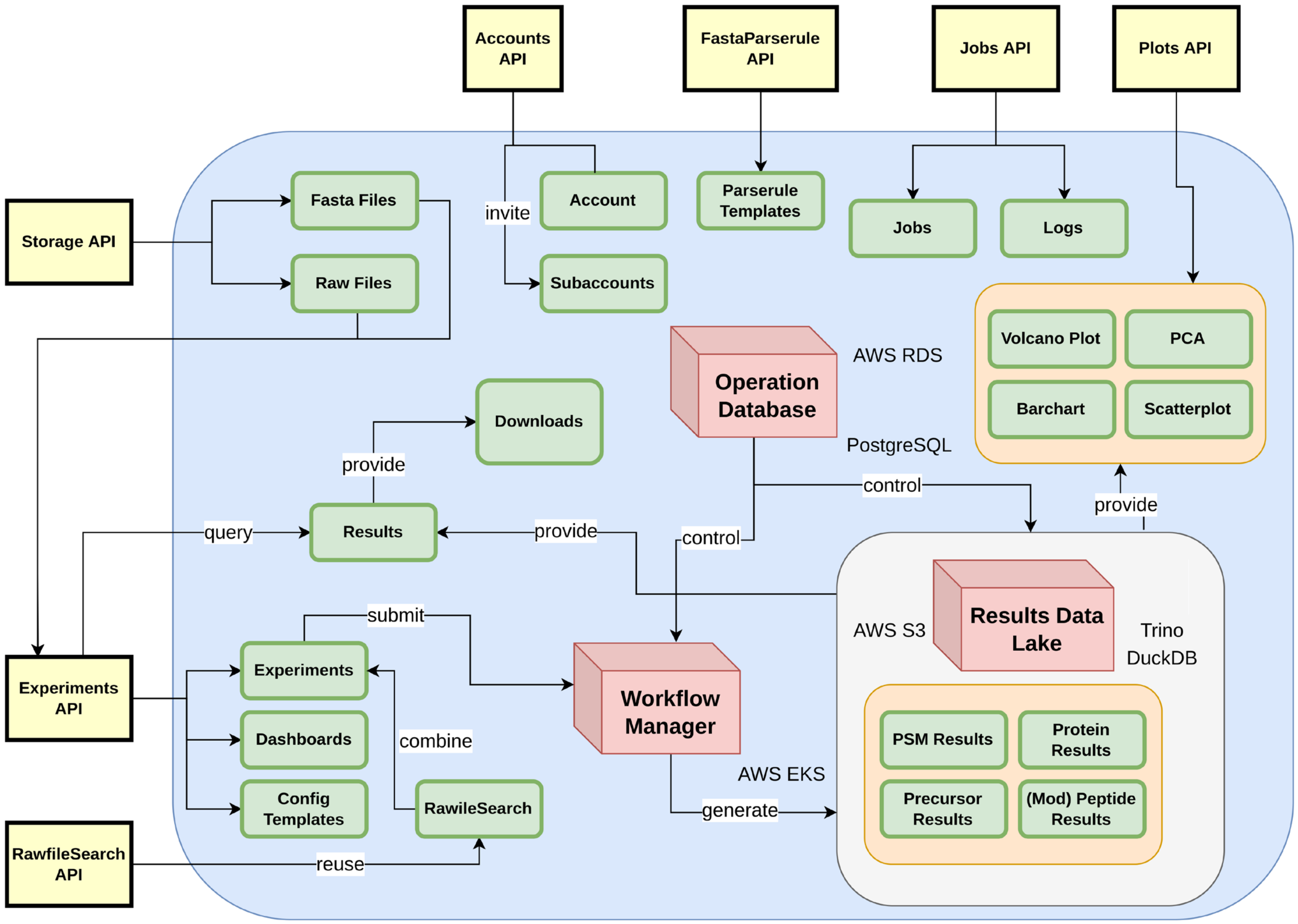

Supporting Figure S1: Structure of the MSAID Platform API. The API is designed to allow construction of proteomics data processing pipelines from intuitive building blocks. It covers the management of users, files, experiments, data processing jobs, and analyses. Both the web and CLI clients rely on the API to present functionality to the user. Alternatively, the user can build their own customized workflows by interacting with the API directly to build a customized workflow. By offering these services as a fully managed API, the complex details of provisioning compute and storage are abstracted away from the user, reducing the cognitive load on the user and enabling efficient scaling of resources for large datasets.
